# Supplementary material for: Saturation genome editing of RNU4–2 reveals distinct dominant and recessive neurodevelopmental disorders
Source: medRxiv. 2025 Apr 10:2025.04.08.25325442. Preprint. [Version 1] doi: 10.1101/2025.04.08.25325442 (PMC12036422; doi:10.1101/2025.04.08.25325442)
Supplement: Supplement 3 [file NIHPP2025.04.08.25325442v1-supplement-3.pdf]

medRxiv preprint doi: https://doi.org/10.1101/2025.04.08.25325442; this version posted April 11, 2025. The copyright holder for this preprint
(which was not certified by peer review) is the author/funder, who has granted medRxiv a license to display the preprint in perpetuity.
It is made available under a CC-BY 4.0 International license .

SUPPLEMENTARY FIGURES

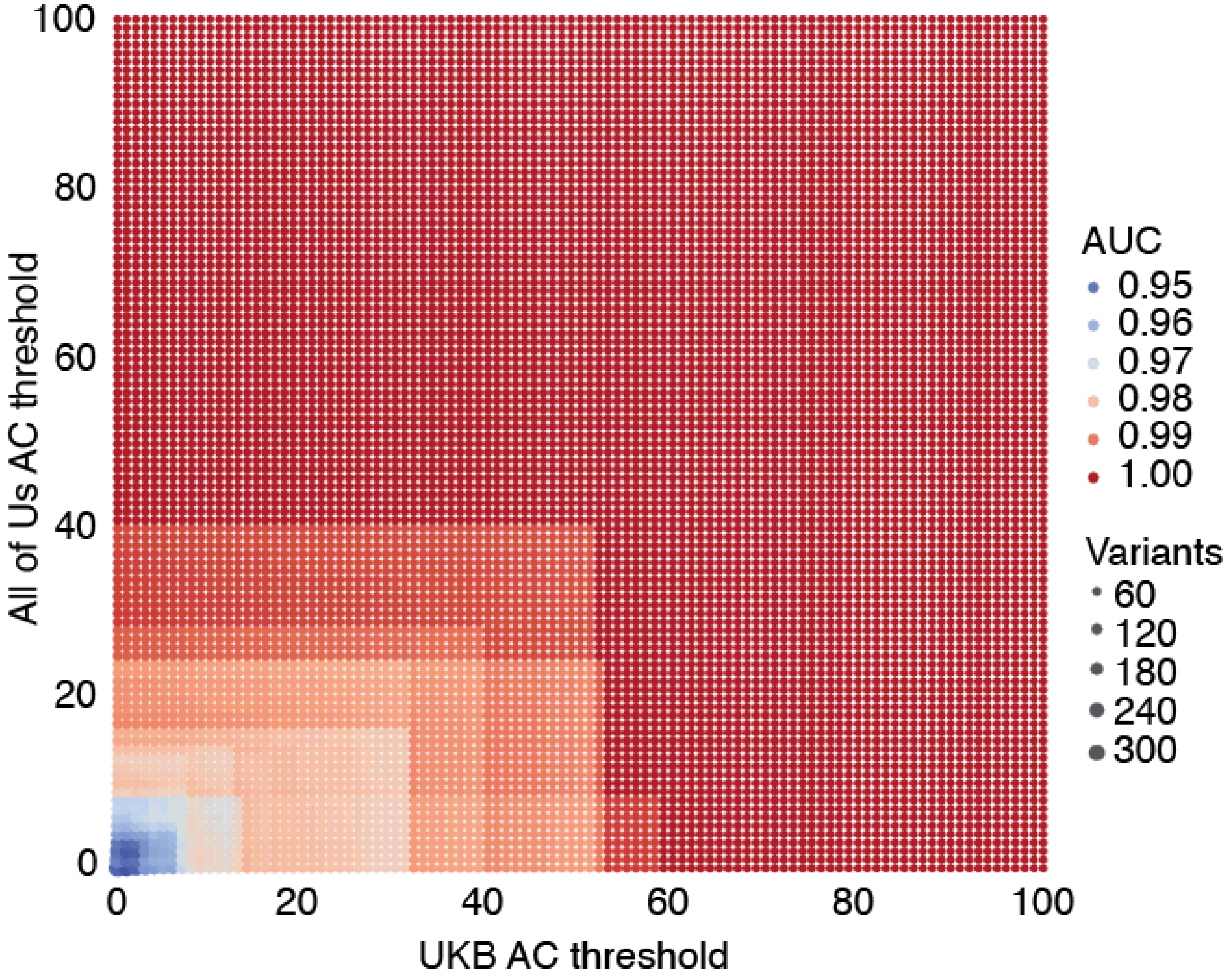

Supplementary Figure 1: ReNU syndrome variants are discriminated with high precision from variants seen frequently in population controls. ROC-AUC measurements for distinguishing 12 ReNU syndrome SNVs from population control SNVs by SGE score are displayed as a heatmap. Each AUC was determined using only variants in UK Biobank and All of Us with allele counts above the thresholds indicated on the axes. Dot size indicates the number of population cohort variants retained in each AUC calculation.

medRxiv preprint doi: https://doi.org/10.1101/2025.04.08.25325442; this version posted April 11, 2025. The copyright holder for this preprint (which was not certified by peer review) is the author/funder, who has granted medRxiv a license to display the preprint in perpetuity.
It is made available under a CC-BY 4.0 International license .

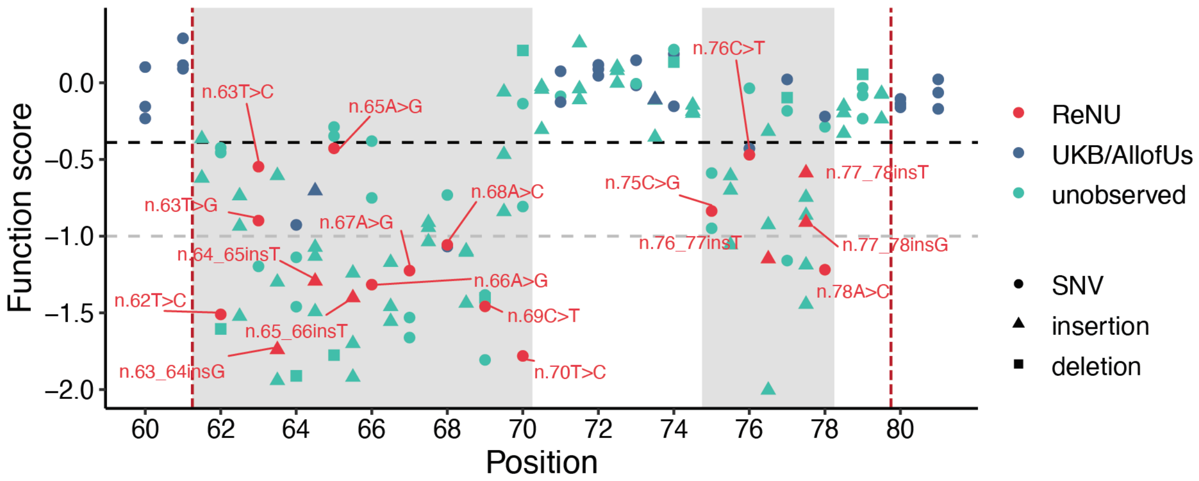

Supplementary Figure 2: Function scores for variants within the *RNU4-2* critical region. Function scores are plotted by position and coloured by their association with ReNU syndrome (red), presence in the UK Biobank or All of Us cohorts (blue), or no observation in either (teal). Variants score lowly in two regions within the CR (shaded), n.62-70 and n.75-78, which correspond to the T-loop and Stem III, respectively. The black dashed line (function score = -0.39) indicates significantly depleted variants and the gray dashed line (function score = -1.00) separates “moderate” from “strong” depletion.

medRxiv preprint doi: https://doi.org/10.1101/2025.04.08.25325442; this version posted April 11, 2025. The copyright holder for this preprint (which was not certified by peer review) is the author/funder, who has granted medRxiv a license to display the preprint in perpetuity.
It is made available under a CC-BY 4.0 International license .

SUPPLEMENTARY TABLES

Supplementary Table 1: Details and function scores for *RNU4-2* variants assayed with SGE. (provided as separate file)

Supplementary Table 2: Comparison of clinical features by function score categories. Two-sided Fisher's tests were used to compare intellectual disability (severe vs mild/moderate), developmental delay (severe vs moderate/mild/none), speech ability (non-verbal/few words vs simple sentences/normal speech), and epilepsy/seizures (yes/one episode vs no) between strong vs moderate depleted variants in the SGE assay. P-values are Bonferroni adjusted for four tests.

Phenotype	Category	Strong	Moderate	OR (95%CI)	*P*-value
**Intellectual disability**	Severe	72/94 (0.766)	1/17 (0.059)	50.4 (7.1-2197.0)	1.45×10^{-7}
	Moderate / mild	22/94 (0.234)	16/17 (0.941)		
**Global developmental delay**	Severe	85/116 (0.733)	1/17 (0.059)	42.7 (6.1-1841.8)	4.28×10^{-7}
	Moderate / mild / none	31/116 (0.267)	16/17 (0.941)		
**Speech**	Non verbal / few words	90/97 (0.928)	1/18 (0.056)	195.5 (24.7-8591.7)	2.66×10^{-13}
	Simple sentences / normal speech	7/97 (0.072)	17/18 (0.944)		
**Seizures**	Yes, more than one episode	68/120 (0.567)	11/19 (0.579)	0.46 (0.11-1.58)	0.829
	One episode	8/120 (0.067)	4/19 (0.211)		
	No	44/120 (0.367)	4/19 (0.211)		

medRxiv preprint doi: https://doi.org/10.1101/2025.04.08.25325442; this version posted April 11, 2025. The copyright holder for this preprint
(which was not certified by peer review) is the author/funder, who has granted medRxiv a license to display the preprint in perpetuity.
It is made available under a CC-BY 4.0 International license .

Supplementary Table 3: **Results from association testing with intelligence-related metrics in the UK Biobank.** Individuals with depleted variants outside of the ReNU syndrome critical region (non-CR; max *n* = 50), and individuals with SNVs with normal SGE function scores (≥ -0.39; max *n* = 12,132) were compared to individuals without variants in *RNU4-2* (fluid_intelligence *n* = 207,458; age left education *n* = 311,233, childhood developmental disorder (DD) = 453,754). The n shown in the table represents the number of individuals in each variant group without missing data that were included in each test.

**Phenotype**	**Variant group**	**n**	**Regression type**	**Coefficient (95% CI)**	***P*-value**
**Fluid intelligence**	depleted non-CR	20	linear	0.366 (-0.523-1.256)	0.420
	not depleted SNVs	5,341		-0.024 (-0.079-0.031)	0.389
**Age left education**	depleted non-CR	34	linear	-0.120 (-0.891-0.652)	0.761
	not depleted SNVs	8,003		0.012 (-0.040-0.062)	0.678
**Child DD**	depleted non-CR	50	logistic	-0.693 (-2.673-1.287)	0.493
	not depleted SNVs	12,134		-0.041 (-0.136-0.054)	0.393

medRxiv preprint doi: https://doi.org/10.1101/2025.04.08.25325442; this version posted April 11, 2025. The copyright holder for this preprint
(which was not certified by peer review) is the author/funder, who has granted medRxiv a license to display the preprint in perpetuity.
It is made available under a CC-BY 4.0 International license .

Supplementary Table 4: Homozygous and compound heterozygous variants in individuals with undiagnosed neurodevelopmental disorders. Equivalent variants in *RNU4ATAC* and their ClinVar classification are included for variants at the equivalent nucleotide and with the same reference (see **methods**). Where a variant is part of a stem region of pairing within the U4 structure, the base it pairs with is noted.

patient(s)	cohort	variant	zygosity	HGVS	function score	region	U4atac equivalent	pairing within U4
1	GEL	chr12-120291785-T-C	hom	n.119A>G	-0.686	Sm protein binding	n.117A>G (pathogenic)	NA
2 and 3 (siblings)	Massimo Mission	chr12-120291859-C-G	het	n.45G>C	-0.719	k-turn	n.50G>C (likely pathogenic)	NA
		chr12-120291785-T-C	het	n.119A>G	-0.686	Sm protein binding	n.117A>G (pathogenic)	NA
4	SeqOIA	chr12-120291785-T-C	hom	n.119A>G	-0.686	Sm protein binding	n.117A>G (pathogenic)	NA
5 and 6 (siblings)	SeqOIA	chr12-120291877-G-C	hom	n.27C>G	-0.900	5' stem loop / k-turn	n.32C>A (not in ClinVar)	n.46 in 5' stem loop
7 and 8 (siblings)	UDN PNW	chr12-120291858-C-T	het	n.46G>A	-0.400	5' stem loop / k-turn	n.51G>A (pathogenic)	n.27 in 5' stem loop
		chr12-120291775-C-T	het	n.129G>A	-0.470	3' stem loop	NA	n.142 in 3' stem loop
9 and 10 (siblings)	Broad CMG / GMKF/ UCSD	chr12-120291872-C-T	hom	n.32G>A	-1.223	k-turn	n.37G>A (path/likely path)	NA
11 and 12 (siblings)	BCM GREGoR	chr12-120291764-C-T	hom	n.140G>A	-0.395	3' stem loop	NA	n.131 in 3' stem loop
13 and 14 (siblings)	Auragen	chr12-120291897-C-G	het	n.7G>C	-0.973	Stem II	NA	NA
		chr12-120291783-A-T	het	n.121T>A	-0.637	Sm protein binding	NA	NA
15	Auragen	chr12-120291897-C-T	het	n.7G>A	-1.123	Stem II	NA	NA
		chr12-120291893-T-G	het	n.11A>C	-0.519	Stem II	NA	NA
16	Auragen	chr12-120291897-C-G	hom	n.7G>C	-0.973	Stem II	NA	NA

medRxiv preprint doi: https://doi.org/10.1101/2025.04.08.25325442; this version posted April 11, 2025. The copyright holder for this preprint
(which was not certified by peer review) is the author/funder, who has granted medRxiv a license to display the preprint in perpetuity.
It is made available under a CC-BY 4.0 International license .

Supplementary Table 5: List of variants in *RNU4ATAC* in ClinVar. The equivalent residue of RNU4-2 was determined for variants in the 5' stem loop / k-turn and Sm protein binding site (see **methods**). Function scores are included where an equivalent nucleotide could be determined and the reference base at that position is the same across *RNU4-2* and *RNU4ATAC*.

U4atac HGVS	Chr	Pos	ClinVar variation ID	ClinVar classification	U4 nt	matching ref?	function score	category	region
n.8C>A	2	121530887	692040	Likely pathogenic					Stem II
n.13C>T	2	121530892	218083	Pathogenic					Stem II
n.16G>A	2	121530895	218082	Pathogenic/Likely pathogenic					Stem II
n.29T>G	2	121530908	977869	Likely pathogenic					5' stem loop
n.30G>A	2	121530909	30180	Likely pathogenic					5' stem loop
n.35A>C	2	121530914	2674599	Likely pathogenic	30	Y	-0.4725	UKB/AllofUs	5' stem loop / k-turn
n.37G>A	2	121530916	218084	Pathogenic/Likely pathogenic	32	Y	-1.2228	UKB/AllofUs	5' stem loop / k-turn
n.40C>T	2	121530919	599282	Pathogenic	35	N			5' stem loop / k-turn
n.46G>T	2	121530925	977856	Likely pathogenic	41	N			5' stem loop / k-turn
n.46G>A	2	121530925	636959	Pathogenic	41	N			5' stem loop / k-turn
n.48G>A	2	121530927	218085	Pathogenic/Likely pathogenic	43	Y	-0.1675	UKB/AllofUs	5' stem loop / k-turn
n.50G>C	2	121530929	30182	Likely pathogenic	45	Y	-0.7194	UKB/AllofUs	5' stem loop / k-turn
n.51G>A	2	121530930	30178	Pathogenic/Likely pathogenic	46	Y	-0.3997	UKB/AllofUs	5' stem loop / k-turn
n.53C>G	2	121530932	30183	Pathogenic/Likely pathogenic	48	N			5' stem loop
n.55G>A	2	121530934	30179	Pathogenic	50	Y	-0.3524	UKB/AllofUs	5' stem loop
n.111G>A	2	121530990	30181	Pathogenic					3' stem loop
n.114G>C	2	121530993	977780	Pathogenic	116	Y	-0.1376	unobserved	3' stem loop
n.116A>C	2	121530995	1373455	Pathogenic	118	Y	-0.7121	UKB/AllofUs	Sm protein binding
n.116A>G	2	121530995	870579	Likely pathogenic	118	Y	-0.8435	UKB/AllofUs	Sm protein binding
n.116A>T	2	121530995	812960	Likely pathogenic	118	Y	-0.0153	UKB/AllofUs	Sm protein binding
n.117A>G	2	121530996	1525441	Pathogenic	119	Y	-0.6861	UKB/AllofUs	Sm protein binding

medRxiv preprint doi: https://doi.org/10.1101/2025.04.08.25325442; this version posted April 11, 2025. The copyright holder for this preprint (which was not certified by peer review) is the author/funder, who has granted medRxiv a license to display the preprint in perpetuity.
It is made available under a CC-BY 4.0 International license .

n.120T>G	2	121530999	977779	Pathogenic	122	Y	-0.6976	unobserved	Sm protein binding
n.124delG	2	121531002	1224468	Likely pathogenic	126	N			Sm protein binding
n.124G>T	2	121531003	2813456	Pathogenic	126	N			Sm protein binding
n.124G>A	2	121531003	39443	Pathogenic/Likely pathogenic	126	N			Sm protein binding
n.23C>T	2	121530902	1133987	Likely benign					loop region
n.41G>A	2	121530920	1629605	Likely benign	36	N			loop region
n.42C>T	2	121530921	1160662	Likely benign	37	N			loop region
n.43A>G	2	121530922	1633266	Likely benign	38	N			loop region
n.45A>C	2	121530924	1632468	Likely benign	40	N			loop region
n.58C>T	2	121530937	1170904	Benign					loop region
n.65C>T	2	121530944	1631270	Likely benign					Stem I
n.87C>T	2	121530966	1168718	Benign/Likely benign					3' stem loop
n.93G>A	2	121530972	1601357	Benign					3' stem loop
n.109T>A	2	121530988	1628512	Likely benign					3' stem loop
n.117dup	2	121530994	1159385	Likely benign					Sm protein binding

Supplementary Table 6. Oligonucleotide sequences used in this study. (provided as separate file)
